# Clinical Phenotyping with an Outcomes-driven Mixture of Experts for Patient Matching and Risk Estimation

**DOI:** 10.1145/3616021

**Published:** 2023-09-13

**Authors:** NATHAN C. HURLEY, SANKET S. DHRUVA, NIHAR R. DESAI, JOSEPH R. ROSS, CHE G. NGUFOR, FREDERICK MASOUDI, HARLAN M. KRUMHOLZ, BOBAK J. MORTAZAVI

**Affiliations:** Texas A&M University, USA; University of California San Francisco, USA; Yale University, USA; Yale University, USA; Mayo Clinic, USA; Ascension Health, USA; Yale University, USA; Texas A&M University, USA

**Keywords:** Cardiology, cardiovascular outcomes, machine learning, medical information systems, mixture of experts

## Abstract

Observational medical data present unique opportunities for analysis of medical outcomes and treatment decision making. However, because these datasets do not contain the strict pairing of randomized control trials, matching techniques are to draw comparisons among patients. A key limitation to such techniques is verification that the variables used to model treatment decision making are also relevant in identifying the risk of major adverse events. This article explores a deep mixture of experts approach to jointly learn how to match patients and model the risk of major adverse events in patients. Although trained with information regarding treatment and outcomes, after training, the proposed model is decomposable into a network that clusters patients into phenotypes from information available before treatment. This model is validated on a dataset of patients with acute myocardial infarction complicated by cardiogenic shock. The mixture of experts approach can predict the outcome of mortality with an area under the receiver operating characteristic curve of 0.85 ± 0.01 while jointly discovering five potential phenotypes of interest. The technique and interpretation allow for identifying clinically relevant phenotypes that may be used both for outcomes modeling as well as potentially evaluating individualized treatment effects.

## 1 INTRODUCTION

Observational medical data, such as electronic health records, present a unique opportunity for analysis of medical outcomes. Primarily focused on risk models, machine learning techniques have leveraged the wealth of data available to generate risk models for adverse event outcomes [29, 39]. However, these techniques are limited in focus, because of challenges in the types of data [8] and the potential for bias from underrepresented samples [32]. As such, studies have been limited in their extension to evaluating treatment effectiveness in outcomes. These studies are primarily limited to results from randomized control trials, with advanced matching techniques needed to compare patients in observational data that may or may not have the same types of data [25].

Because randomized control trials are often costly, homogenized, and time-consuming, techniques are needed to allow for the comparative effectiveness of observational data. These techniques cannot solely observe clinical risk, because the observed treatment effectiveness heterogeneity has numerous causal inference implications and challenges [9, 40]. An alternative approach, then, is to use propensity matching techniques to draw comparisons in observational data [3]. These techniques model the likelihood of a treatment being prescribed from the data provided, to properly identify those that could be randomized to different treatments versus those that would clearly benefit or are clearly contraindicated from such treatments [26]. Such techniques were used to perform observational comparative effectiveness of **mechanical circulatory support (MCS)** devices in a population of patients suffering from **acute myocardial infarction (AMI)** complicated by **cardiogenic shock (CS)** [5]. Briefly, CS is a deadly complication of AMI, where mortality and other major adverse events (i.e., major bleeding) remain high despite a number of advances in technology aimed at treating AMI-CS. A series of MCS devices, known as intravascular microaxial **left ventricular assist devices (LVADs)** were compared against **intra-aortic balloon pumps (IABPs)** to assess device effectiveness [6] in light of increased utilization of LVADs since their introduction to the market [7]. However, authors note a potential limitation in the matching scheme, because propensity matching techniques may provide similar clinical phenotypes, however, homogeneous subpopulations within the heterogeneous cohort may exist that have the opposite relationship with outcomes [10]. Because propensity matching techniques do not consider risk factors and outcomes directly, limitations to their findings exist.

A technique that jointly learns clinical phenotypes (subpopulations) that also jointly learns the risk of major adverse clinical events (i.e., mortality), is needed. Such a technique would be better suited to identifying subtypes of patients that may have varied individual treatment responses that differ from the average treatment effect. This would then be hypothesis-generating for future randomized control trials, identifying different test criteria for each group of patients. This work aims to develop such a jointly learned model, to model patient outcomes as well as identify clinical phenotypes that may contribute to treatment heterogeneity, thus overcoming the limitations to propensity matching techniques [9]. To accomplish this objective, we employ a deep **mixture of experts (MoE)** model [13, 16, 35] to jointly identify clinically meaningful phenotypes and to risk stratify patients. Using the data and cohort defined by Dhruva et al. for AMI-CS [6], this article aims to jointly learn the risk of mortality and discover phenotypes in which treatment effectiveness can be evaluated. The potential outcomes of this work are hypothesis-generating findings that may lead to future studies of treatment effectiveness and trial design using IABPs and LVADs.

A preliminary version of this work has been reported [16]. This article represents a substantial extension of these preliminary findings. This work explores the latent space that is created by the models, to provide further evaluation and interpretation of predictive results and contextualize false positives and false negatives. In addition, the preliminary work was free to select any number of components. This work compares a regularization technique to limit the choice of components (clusters) in a data set and further examines limiting the number of components to a size-restricted method in which each cluster has enough cluster membership. Cluster membership has been noted as a limiting factor in other cardiovascular phenotyping work because of concern with respect to the number of patients within a cluster and the ability to model that cluster’s outcomes [1]. Finally, we consider a broader suite of evaluation metrics. The contributions are as follows:

- Developed an MoE approach for phenotype discovery when modeling for risk of mortality in patients with CS.
- Developed a method by which patient phenotype discovery can be regularized.
- Allow for a soft-clustering approach for multiple phenotype contributions.
- Visualize and interpret the latent clustering space to explain model discovery and performance, jointly.
- Evaluate performance metrics and clinically relevant calibration [37] on models.
- Evaluate performance metrics when the number of components is freely chosen versus when clusters are required to contain a certain percentage of population data to be considered.

The rest of this work is organized as follows: Section 2 reviews related work, while Section 3 discusses the MoE technique, Section 4 discusses the experimental setup and findings, Section 5 reviews limitations and future directions, while Section 6 concludes this work.

## 2 RELATED WORK

### 2.1 Clinical Phenotyping

Discovering phenotypes of specific conditions may allow for improved understanding of the process and trajectory of diseases, treatments, and outcomes [23]. Deep learning techniques, for example, sparse autoencoders, provide data-driven strategies to phenotype, embedding patient data into latent spaces [20] and then visualized by **t-distributed stochastic neighbor embedding (t-SNE)** [22] to identify novel subpopulations. Other embedding and visualization techniques used **uniform manifold approximation and projection (UMAP)** [27] followed by Gaussian mixture modeling to cluster emergency room patients and their chief clinical complaints [17], while others expanded on using UMAP to identify phenotypes of COVID-19 using X-ray images [18]. Most work, however, describes phenotypes by risk, or clustering without knowledge of outcomes-driving risk factors. Semi-supervised techniques that combined denoising autoencoders and random forest classifiers to discover phenotypes of patients with amyotrophic lateral sclerosis have also shown to have success [2], and this work takes such an approach to phenotype discovery in jointly learning risk models.

Deep learning can aid in modeling causal effect inference [21, 41]. One such approach used variational autoencoders and inferred causal structure within the latent space [21]. Other work used latent representations to identify similarities in patients for estimating treatment effect [41]. Several techniques have recently been used for finding phenotype clusters using risk profiles. Chapfuwa et al. proposed a novel approach that involved clustering patients in a latent space and differentiated clusters by different time-to-event risks [4]. Their clustering was performed with a joint learning of cluster assignments and time-to-event predictions. Nagpal et al. used a generative mixture model for inferring treatment effect differences in patients treated with opioids and whether or not patients would still be using opioids one year after initial treatment [30]. These works provide a foundation for similar approaches in risk outcomes modeling with CS patients. We similarly jointly learn cluster assignments and outcomes predictions to infer treatment effect differences within the discovered subgroups.

### 2.2 Deep Mixture of Experts

The concept of using a deep MoE for supervised learning was first proposed in the early 1990s [19, 31]. In their earliest formulation, these MoEs were each simple feed-forward networks and the gating function a softmax function over those outputs. More recently, the concept has attracted a great deal of attention in applications involving large neural networks for a variety of tasks, such as language modeling [35], activity recognition [13, 36], and image recognition [38]. MoEs provide many modeling advantages and limitations [24]. One area they have demonstrated improvement in performance is in health data, where subpopulation-specific analyses are conducted. Indeed, because of their ability to divide-and-conquer the patient dataset, MoEs are capable of modeling outcomes for patients that may have differing risk factors [11, 15, 33]. MoEs are capable of dividing and conquering the prediction space with any downstream classifier, so long as it is a differentiable function, making neural networks a good candidate for implementation [34].

Shazeer et al. [35] used an extremely wide MoE between stacked **long short-term memory (LSTM)** layers for language recognition and described how sparse gating of their model allowed for decreased overall computational cost. They described several challenges faced in the training of MoE-based models, namely, the problem of batch sizes effectively shrinking as gating functions are learned and the challenge of balancing expert contributions. The shrinking batch problem can be addressed by keeping the number of experts small, by increasing batch size in proportion to the number of experts, or by taking advantage of data structure via convolutions. In this work, we keep the number of experts small, with the primary goal to improve clinical interpretability, but with the secondary goal of addressing this problem. Shazeer et al. balanced expert contributions through the use of a loss function penalizing unequal expert importance [35]. Additionally, event rate limitations and a different set of collected risk factors that lead to low and high risk for different patients may limit MoEs [14], discussed further in Section 5; however these limitations do not impact our work here as the subpopulations sought in this work all have the same complete data set.

## 3 METHODS

This section discusses our deep mixture of experts methodology, in which we sought to: (1) design a supervised learning problem to predict major adverse clinical events (mortality), and (2) find potential phenotypes by clustering patients through expert activation. By coupling these objectives, we formulate a semi-supervised problem where a clustering schema can be learned as part of the supervised problem, but separated from the supervised case for the classification of new patients prior to treatment. In this work, information is learned in three tiers: initial data, data following treatment, and outcomes. The initial data contain information that could be useful for identifying how to treat a patient. Once treatment has begun, a second tier of additional information is available, but as this information was influenced by treatment choices, it should not be used for clustering. Finally, after treatment, outcomes occur. While no direct causal inference is made here, we cluster prior to treatment information and predict outcomes with all data. Therefore, this work provides a framework for future causal inference and individualized treatment effect estimation by learning across data gathered at different times throughout a patient’s hospitalization: (1) The data available from arrival in the emergency department to prior a procedure and treatment decision making (hereafter presentation data); (2) The data captured about treatment decisions during intervention including use of MCS devices (hereafter treatment data); and (3) ultimately, in-hospital outcomes data (in-hospital major bleeding, in-hospital mortality).

**Fig. 1.**
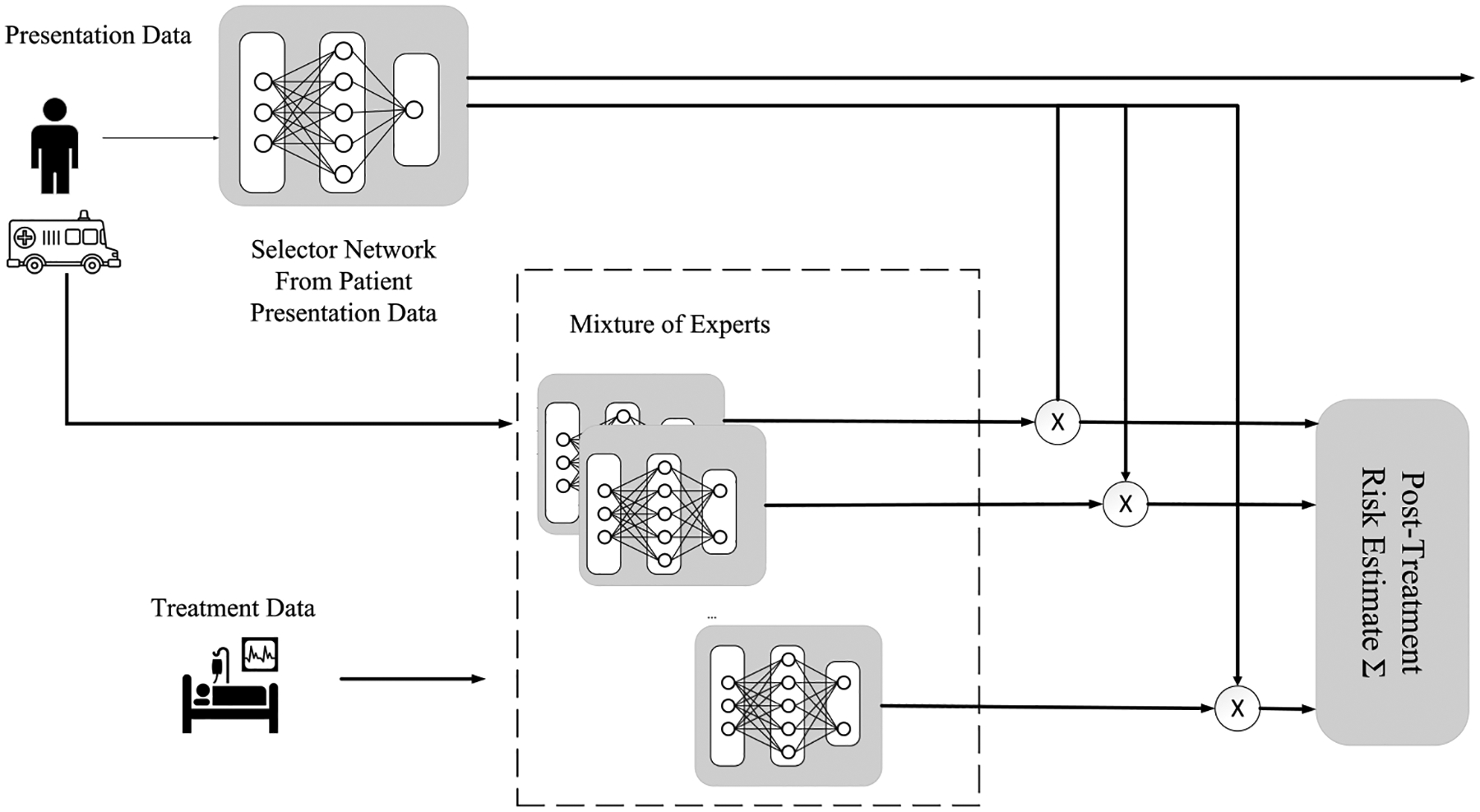
Deep MoE model for clustering and predicting clinical outcomes. The initial selector network takes the data available at patient presentation, while the expert networks make their risk estimates based on presentation and treatment data. The final estimate is soft-weighted voting across experts as determined by the selectors.

The overall architecture is illustrated in Figure 1. The deep mixture of experts is divided into two key components—the selector-network and the mixture of expert-network networks. The selector-network network is provided with presentation data and provides two outputs, some initial prediction of outcomes as well as a softmax distribution of probabilities of how many clusters the participant may belong to (i.e., how many expert networks should be activated). We search the *n* clusters with a hyperparameter, discussed below in Section 3.2. In the second stage, the series of expert-network make predictions based upon the presentation data and treatment data, where each individual network makes the prediction of an outcome. The initial network description and training are presented in Reference [16]—and what follows is a detailed review of the network training as well as additions to the phenotype discovery, model regularization, and latent space analysis.

### 3.1 Deep Mixture of Experts

The input to the selector-network (first tier of information) is passed through a number of fully connected layers. The final fully connected layer is passed on to each expert-network, and is also fed into a gating layer with size equal to number of experts. Each expert-network concatenates its input (second tier of information, treatment information and later) to the pass-through output of the selector-network, and then this is fed through several fully connected layers. The final layer of each expert outputs an outcome prediction. Each of these predictions are weighted by the selector-network’s gating layer and summed for the final model prediction. The model as used here harnesses this MoE approach for supervised outcome prediction with expert activations serving as the unsupervised cluster assignments. Models were implemented in Tensorflow 2.3.2. We evaluated five configurations of model architectures that varied the network sizes and the network connections between the selector-network and expert-network networks. First, in the largest configuration, each network is the same size with three fully connected layers of 50 nodes each layer. The middle configuration reduces to only two connected layers and only 24 nodes. The smallest network uses only a single layer for the expert-network expert prediction. Because of the nature of the registry data used (described further in Section 4.1), the fully connected networks were sufficient in complexity without risking overfitting, as in the case of convolutional neural networks and larger structures. We also tested two configurations of the selector/expert-network. In the first configuration, the selector-network provides only a gating mechanism, whereas in the second version the selector-network provides an initial risk estimation that is provided to the expert-network to “update” after receiving treatment information data.

### 3.2 Number of Experts

The advantage of the MoE architecture designed is the ability to jointly cluster and produce risk estimates. This joint clustering, however, requires prior knowledge on the number of clusters it is free to assign to. In other words, the architecture must know the number of expert networks to design and training data to be fed to each expert network. However, as the number of experts increases, the batch size must also increase, or the training will suffer from the shrinking batch problem [35]. We avoided this problem by constraining the number of experts: a large number of experts would be difficult to interpret, and our clinical condition is focused, therefore a large number of phenotypes discovered may be too small (too few individuals) to consider it. Therefore, we limited the number of experts (clusters) to 10 to aid future clinical utilization of the phenotype discovery.

### 3.3 Model Regularization

A chief difficulty in building and training this model lies in the appropriate choice of regularization for the selector-network probability outputs. In underregularized setups, this model collapses to use only a single expert, while in overregularized setups the model uses all experts evenly, but does not consistently group any subjects together. L2 regularization penalties were applied to the output of the selector-network. These penalties encourage an even utilization of all experts, allowing the overall model to selectively use specific experts for classification.

### 3.4 Latent Space Analysis

**Fig. 2.**
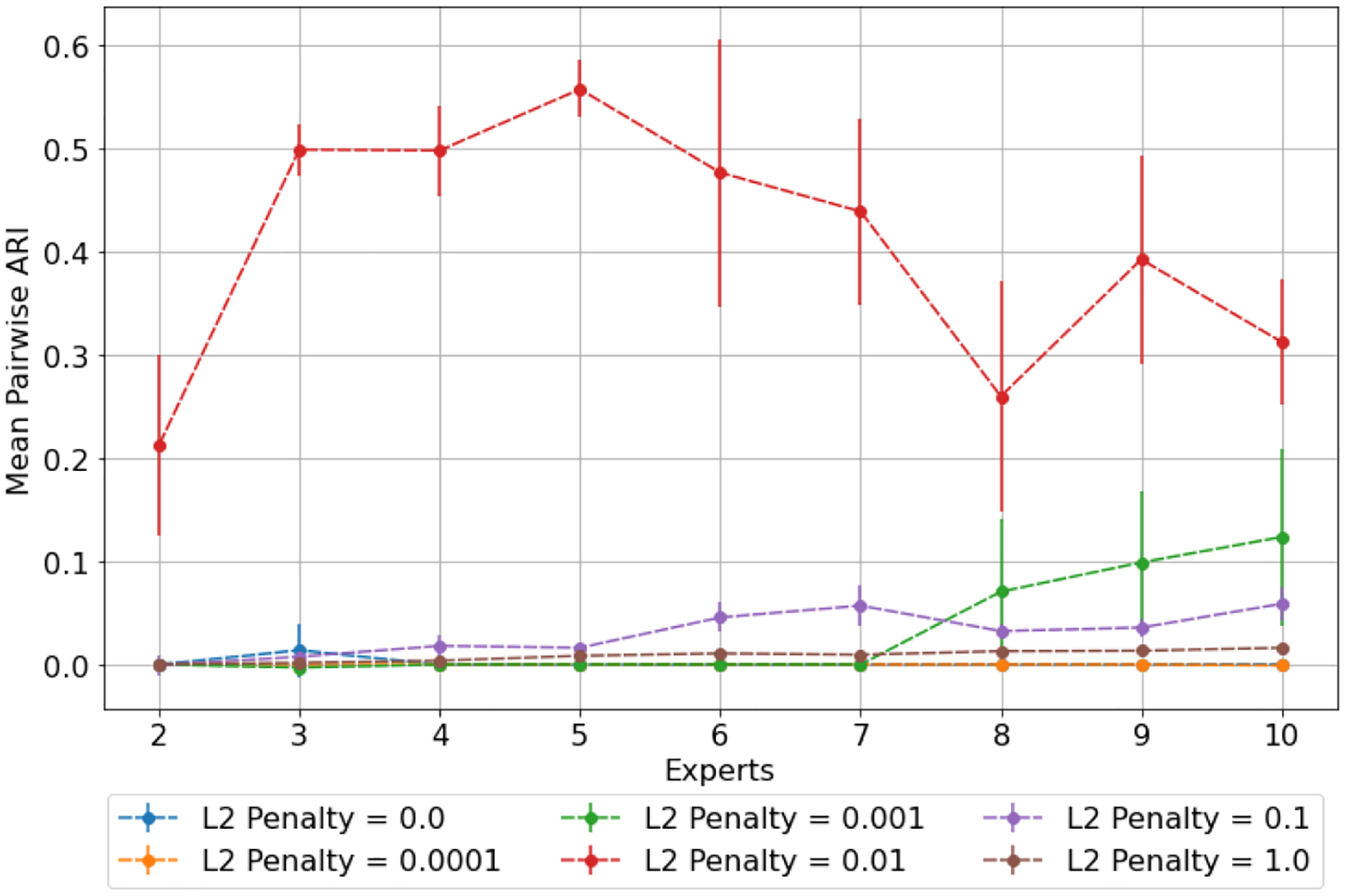
Mean pairwise ARI given *n* experts and various L2 penalties in the clinical dataset. Confidence bars express 95% CI. Reprinted, with permission, from Reference [16]. ©2021 IEEE.

The latent space produced by the gating layer is a key point of interest. The softmax output of that layer scales all outputs collectively to sum to 1. This results in many outputs being either very close to 0 or very close to 1, but some subjects have less extreme weightings assigned to their experts. We observed that even though the optimal performance was achieved with three to five experts (Figure 2), some number of experts contain very few assignments (Table 5). As noted in the literature [1], this can have a limiting factor on modeling the relationship between patients within a cluster and their outcomes. As a result, we compare two approaches to evaluating the latent space and visualizing findings. First, we present our findings for both models with three experts and models with five experts. Three experts were chosen to ensure no cluster had less than 10% of the population of the largest cluster, as well as ensuring the weight contribution of each cluster to the final model was greater than 10%. Then, we visualize the latent space of the selector-network’s gating layer using a ternary plot. This allows us to visualize the three experts that contribute the strongest (with five experts) against a visualization where the model has only three experts. With this approach, we visualize the three experts that contribute most strongly to the output, and we truncate beyond three experts. We align the expert assignments so that the expert receiving the greatest overall weight is always shown in the bottom right of these plots (labeled “A”), the expert receiving the second most weight is shown to the top (labeled “B”), and the expert receiving the lowest weight is shown to the bottom left (labeled “C”). Points in these plots are colored based on their predicted label using a simple 50% prediction threshold with respect to the outcome prediction (True/False Positive/Negative). To assess the model performance and uncertainty as a function of the latent space, we generated heatmaps with local AUROCs and calculated as the AUROC of all samples within the given grid space.

### 3.5 Training, Validating, and Testing: Baseline Models and Performance Metrics

We compare against standard clinical baselines (logistic regression with L2 regularization) and against XGBoost, a nonlinear classifier for modeling higher-order, nonlinear relationships. We hyperparameter tune the regularization parameter for logistic regression, and we hyperpameter tune XGBoost (learning rate: 0.3, maximum depth: {1,6}, and number of trees: {50,500, 50 step size}—selecting 0.3, 6, and 100, respectively). We report model performance using the **area under the receiver operating characteristic curve (AUROC)**, and we use the **adjusted Rand index (ARI)** for evaluating the similarity of cluster partitions over the folds [12]. ARI is used to assess clustering stability between different model folds, where zero indicates that any similarity in the partitioning is likely due to chance and one indicates similar clustering. We also evaluated secondary metrics, including the **area under the precision recall curve (AUPRC)** and the Brier Decomposition, through calculation of the uncertainty, resolution, and reliability for clinical calibration of the model [37]. While these metrics were not used primarily for model parameters, they were noted if they varied by greater than one standard error compared to other metrics for component selection. Models were trained, validated, and tested in a five-fold cross-validation. For each iteration, the training-fold then had 20% of the data split for internal validation for hyperpameter tuning of the model as well as the regularization parameter. We conducted a grid search for the L2 penalty from 0.001 to 0.1 ({0.001, 0.002, 0.004, 0.006, 0.008, 0.01, 0.05, 0.1}) as well as varied the number of defined experts to find the optimal model size and number of components (0.01 was determined to be the optimal penalty). All results reported here reflect the mean ±95% confidence interval over the cross validated results.

## 4 EXPERIMENT AND RESULTS

### 4.1 Clinical Data

The approach was modeled on data derived from the Chest Pain-MI registry and the CathPCI Registry from the National Cardiovascular Data Registry provided by the American College of Cardiology, as in Reference [6]. These registries represent the highest quantity of well-curated, complete, quality clinical data (extremely low rates of missing or noisy data) from over 1,500 clinical sites across the United States [28]. Binary variables were imputed as “no” for indicator variables and mean was imputed for continuous variables. The Chest Pain-MI registry captures detailed information on presentation characteristics for individuals presenting with AMI, regardless of those undergoing PCI, a first-line treatment for patients with AMI. The details of the procedure itself are captured in the Cath-PCI registry, which includes granular data on periprocedural care and procedural characteristics, thereby complementing the information available across registries. As a result, this dataset represents reliable clinical data on individuals with cardiovascular disease that may also undergo a cardiovascular procedure (e.g., PCI), with extremely low rates of missing data or outliers due to data noise, well-suited for modeling. This study was approved by human research protection programs (TAMU IRB #2018-0856 and Yale University IRB #0607001639).

First described in References [6, 16], this dataset contained 28,304 patients that suffered from an **acute myocardial infarction complicated by cardiogenic shock (AMI-CS)**. From the data available from the two registries, we selected twelve features deemed clinically relevant for potentially phenotyping the patients and fifteen additional features that were deemed likely to be clinical risk factors for mortality prediction, as selected by board-certified cardiologists, and these variables are listed in Table 1.

### 4.2 Mortality Estimations

The final model produces a probability estimate of an outcome occurring. This estimate is informed by decisions made at treatment time and information learned after treatment, and so should not be used for describing patient phenotypes. Therefore, the full risk estimation is polluted by the knowledge of the future (data leakage) and is not appropriate for use in describing clinical phenotypes in a way that would be useful prior to treatment. The risk estimation model provides increased utility than a standard risk model by providing insight at two key decision points. First, at the time of treatment, the model provides an initial risk estimate given pre-treatment covariates, which can inform subsequent clinical interventions to reduce risk. Second, after assigning treatments and determining clusters that patients fall within, the final risk model provides a risk of mortality, with cluster-specific features. This allows the phenotype-driven model to provide differing risk factors for model importance across varying clusters and patients the model is trained on, which aids in phenotype identification and model interpretation, which may contribute to the interpretation of treatment heterogeneity with respect to outcomes.

An alternative approach to this problem is to train two separate models, a risk estimation model that incorporates pre-treatment and treatment variables and a clustering model that focuses only on clustering patients prior to treatment and without any use of future information. Indeed, several research efforts have found promising patient clusters through this approach (See Section 2.1). However, the key benefit of our multilayered MoE approach is that the training of the model drives the selector-network to extract features that are associated with the treatment and outcome. Rather than finding clustering boundaries on the most numerically distinct features, this approach allows for clustering driven by features most associated with the outcome.

The results of the mortality prediction are shown in Table 2, originally from Reference [16]. Briefly, this table demonstrates the accuracy of baseline models versus the MoEs employed here. Note that the smaller models perform better, likely due to the tabular nature of the clinical data. Given comparable risk estimation performance, then, the small and medium-sized MoEs are then able to provide phenotypes in which risk factors and treatment differences may vary in meaningful and comparable ways, and we use the small models moving forward. The results are shown with an L2 penalty of 0.01.

In this work, we extend our preliminary results with a broader hyperparameter tuning algorithm, in particular with L2 penalties. The process remains the same from Reference [16] and the findings are the same. We ran this as a two-stage process of evaluating the mean pairwise ARI and then the mean AUROC based on a search of L2 penalty parameters. Table 3 lists these values for the small model. We note that pairwise ARI suggests that any L2 value between 0.004 and 0.01 has significance over the other L2 penalties. In addition, it suggests five experts as the ideal number of experts as the peak value across the L2 penalties (with the exception of 0.004, which suggests six experts). With near consensus across the penalties, we selected five experts for our model. Then, we looked at AUROC for predictive value to select the final L2 penalty, where the maximum AUROC comes with L2 = 0.01 (with either four or five experts). From this examination, we concluded that five experts and an L2 penalty of 0.01 was ideal across metrics. We note that these results are updated from Table 2, and perhaps more importantly show robustness in both model performance and component selection across random restarts of cross-validation.

We additionally looked at the findings of the small model with respect to the AUPRC and the Brier Decomposition results. Those results are shown in Table 4. Briefly, as with AUROC, a higher AUPRC is desirable. The Brier Decomposition provides a series of measurements that provide for model calibration. In brief, they present how well predicted probabilities of an event match observed probabilities of that event. One would seek reduced uncertainty measures the rate of the outcome, and model calibration is then defined by the resolution and reliability, where higher resolution and lower reliability scores indicate better model calibration. We refer readers to Reference [37] for additional information on these results. Uncertainty is unchanged with L2 as it measures the event rate. We see that the resolution remains fairly consistent across all L2 penalties, with higher values coming in the 0.006 to 0.01 range, though there are some of the highest values at 0.001. However, the reliability is better with is best with 0.008 or higher. These findings all suggest L2 penalties between 0.006 and 0.01 would perform best, consistent with our primary findings using ARI and AUROC.

Figure 2 plots the pairwise ARI, across each fold, of the best performing model: the small model. As a single monolithic cluster is undesirable, the ARI was set to 0 for any pairings involving a singular clustering. There is high inter-fold cluster stability, particularly when a modest regularization parameter is employed (l2 penalty of 0.01) for five clusters. A model with three clusters is similarly stable and potentially more interpretable and the two results are compared further.

In the case of the five-cluster model, there was consistently one large cluster (A), one outlier cluster (E), and three intermediate clusters (B–D). The features for each of these clusters is provided in Table 5. The largest cluster tended to contain older patients, more of whom tended to be female and had a higher mortality rate. The intermediate clusters had lower mortality rates with higher rates of MCS utilization (B) and differed from the other intermediate clusters (C and D) through presentation conditions of the AMI-CS: different status of PCI and single vessel disease as opposed to multi-vessel disease–likely leading to increased utilization of the IABP or LVAD. The small cluster E demonstrated potential outliers of individuals that did not fit a given phenotypic description and require further study.

Table 6 provides the mortality rates for those clusters. This table is shown with 95% confidence intervals as calculated by the Clopper-Pearson method.^1^ Given MCS utilization, as was the focus of prior work [6, 7, 16], MCS utilization was associated with higher mortality (likely prescribed for patients observed to be at greatest risk), and a mortality difference appears to be associated with the choice of IABP versus LVAD, consistent with prior findings, but varied amongst the clusters.

### 4.3 Latent Space Findings

To explore the selector-network latent space, two models were selected for further analysis, one constructed with three experts and one with five. From among these two models, the majority of cluster weight was contributed by three of the experts present, as noted in Table 7, allowing for visualization on a ternary plot.

**Fig. 3.**
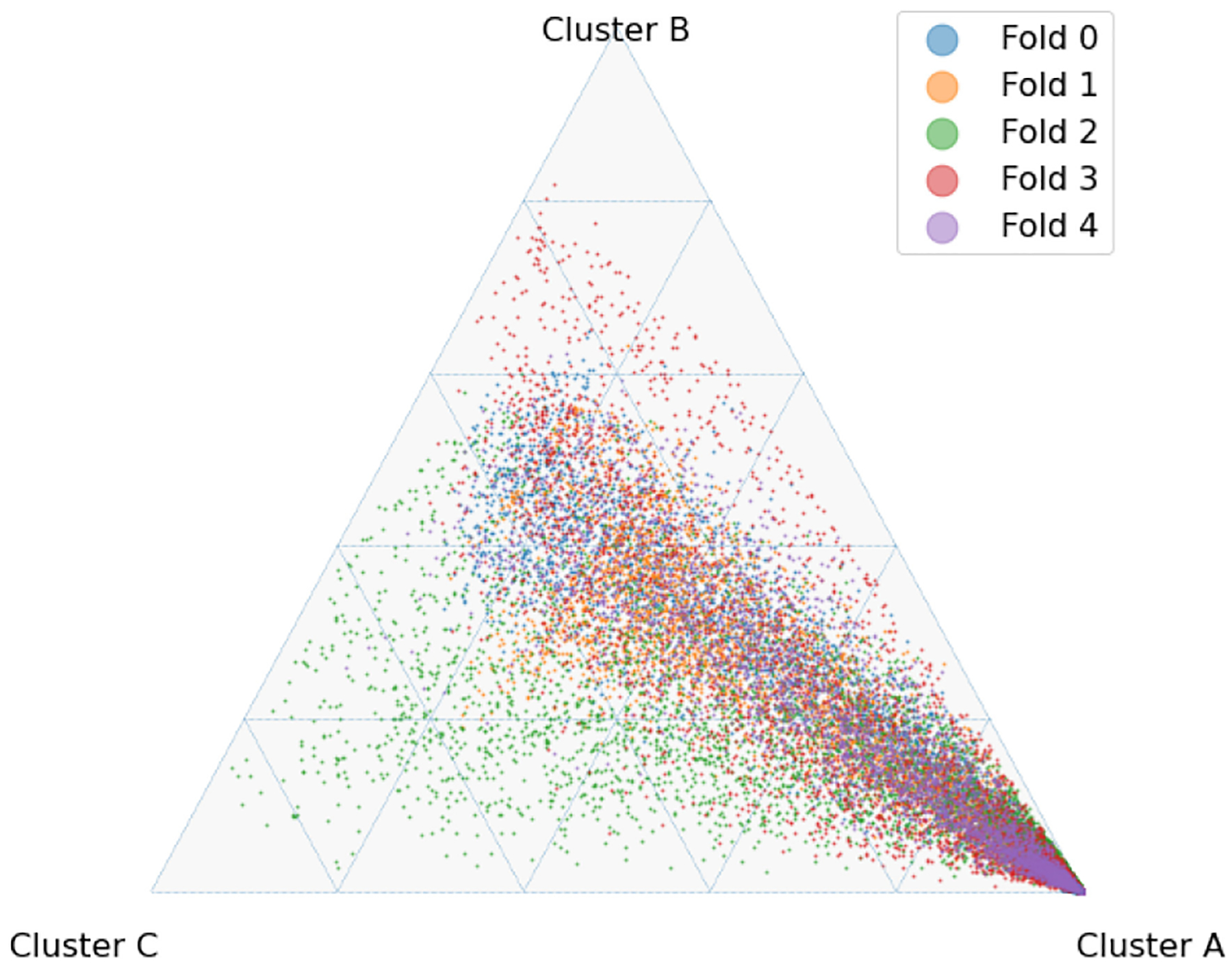
Ternary plot of selector-network output with five experts and L2=0.01.

**Fig. 4.**
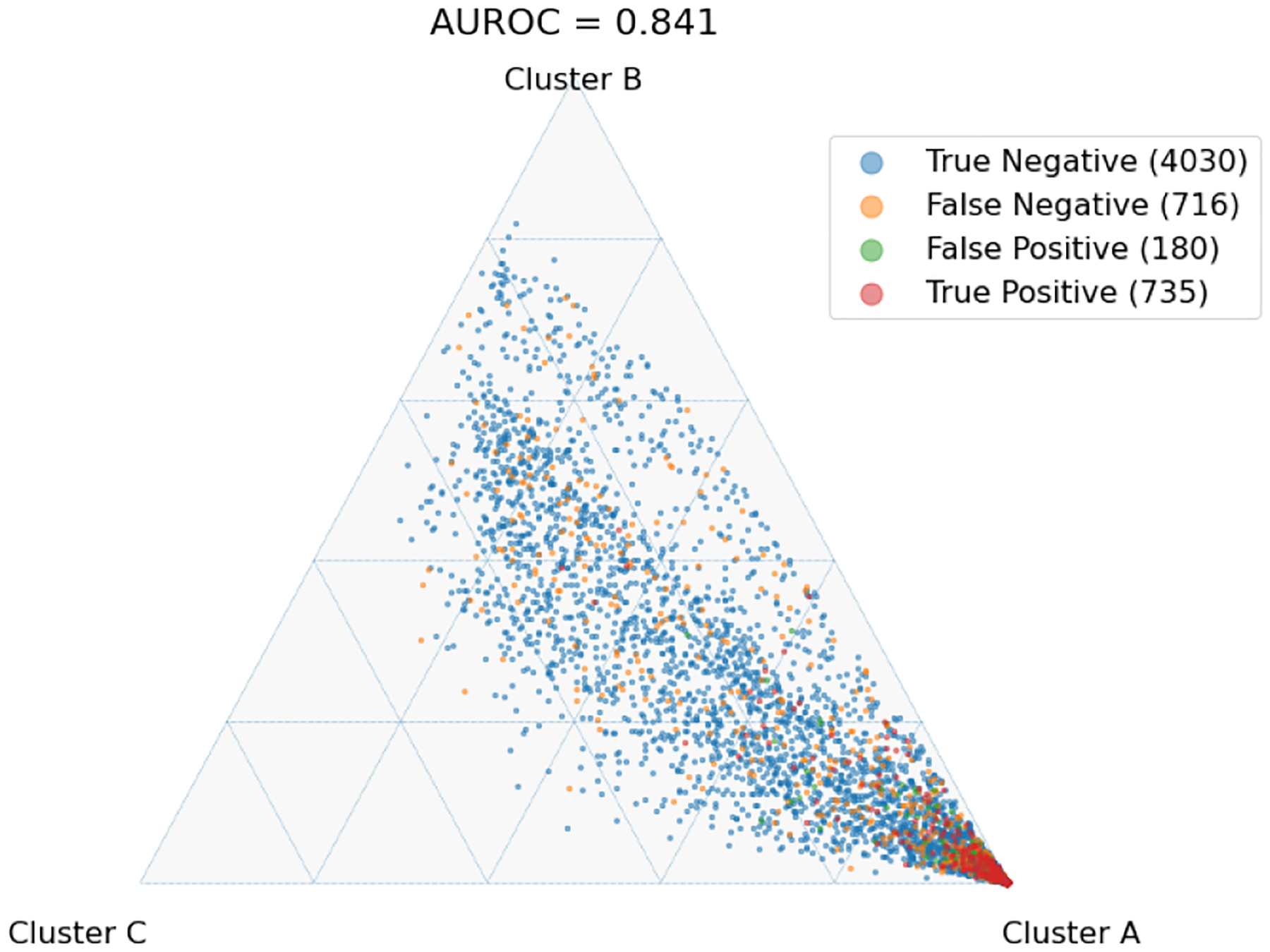
Selected single-fold ternary plot with five experts and L2=0.01. Colors indicate prediction and correctness assuming a simple 50% threshold.

**Fig. 5.**
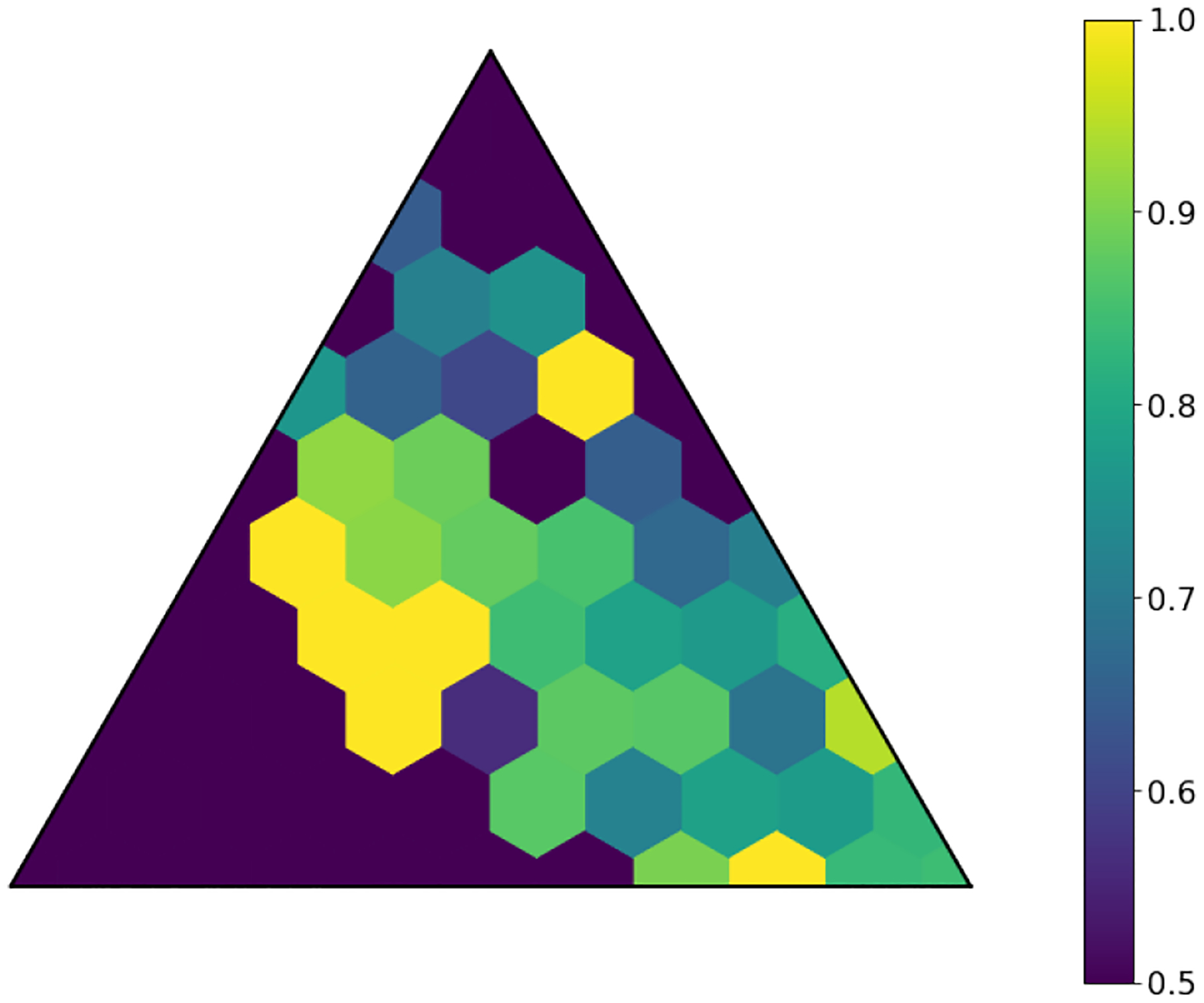
Local AUROCs of model with five experts and L2=0.01. Cells with insufficient subjects for scoring are set to 0.5.

**Fig. 6.**
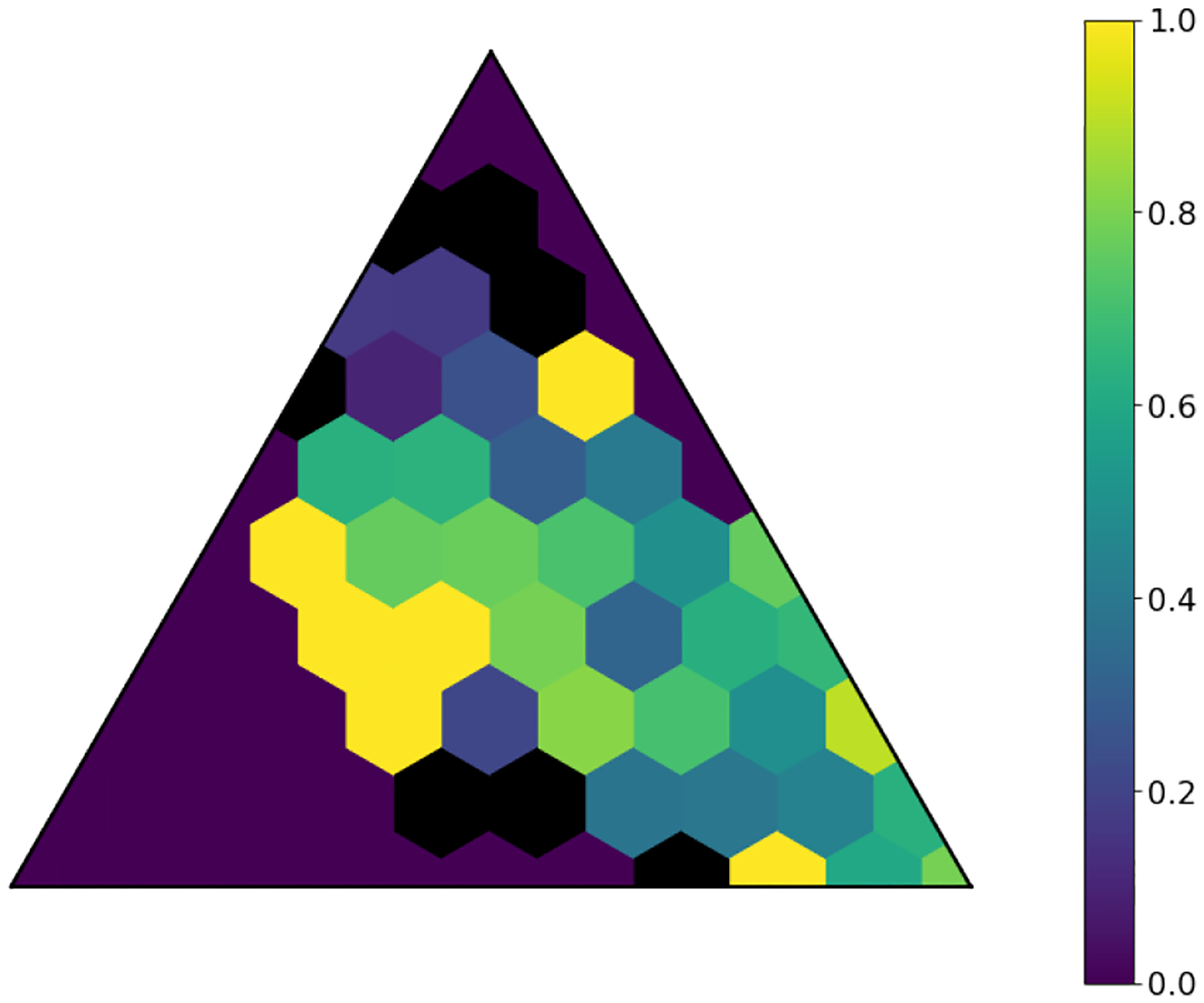
Local AUPRCs of model with five experts and L2=0.01. Cells with insufficient subjects for scoring are set to 0.

For the model with five experts, the distribution of patients among each fold is shown in Figure 3. Two experts are truncated. However, their combined impact on outcome prediction is minimal: one expert supplies a total of 2.1% weight of all experts, while the other supplies less than 0.1% of that weight (Table 7). Cluster A can be seen to be the most weighty cluster, contributing 77% of all weight. A representative fold from this model is shown in Figure 4. In this figure, true positives are found more often near Cluster A, while subjects more distributed throughout the other clusters exhibit lower overall risk. Local prediction performance is shown in Figures 5 and 6. Prediction quality can be seen to improve among patients who are more balanced between clusters.

**Fig. 7.**
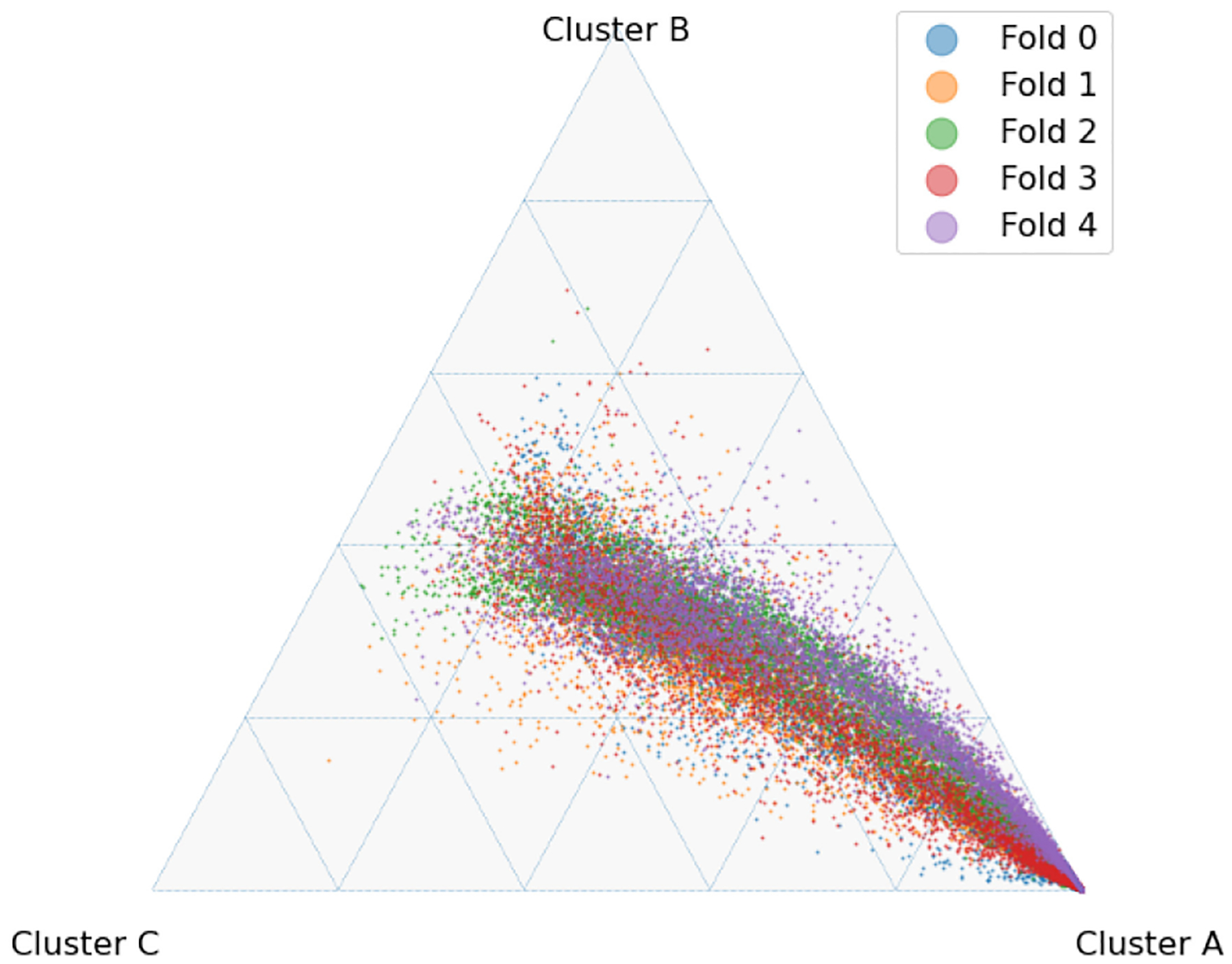
Ternary plot of selector-network output with three experts and L2=0.01.

**Fig. 8.**
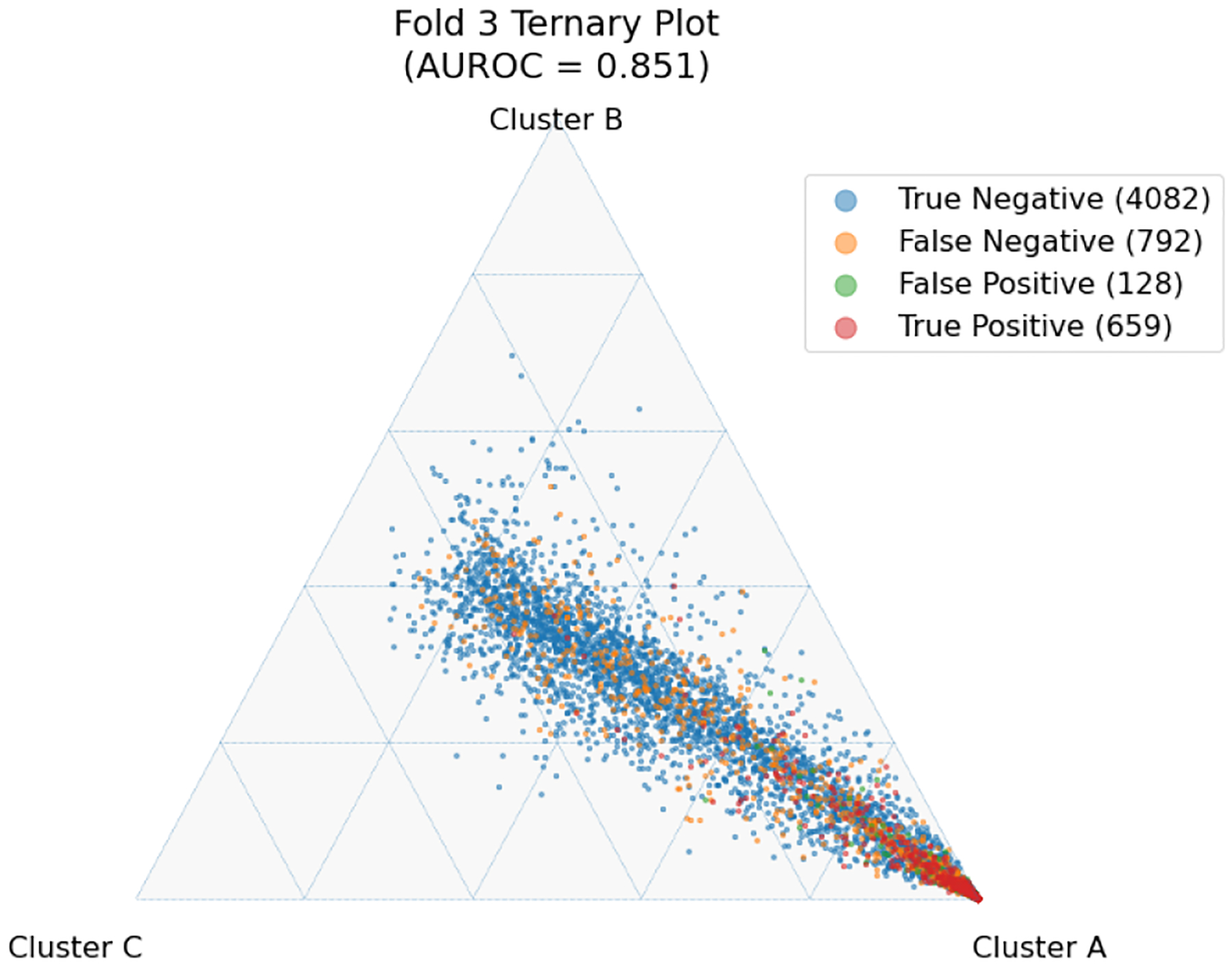
Selected single-fold ternary plot with three experts and L2=0.01. Colors indicate prediction and correctness assuming a simple 50% threshold.

**Fig. 9.**
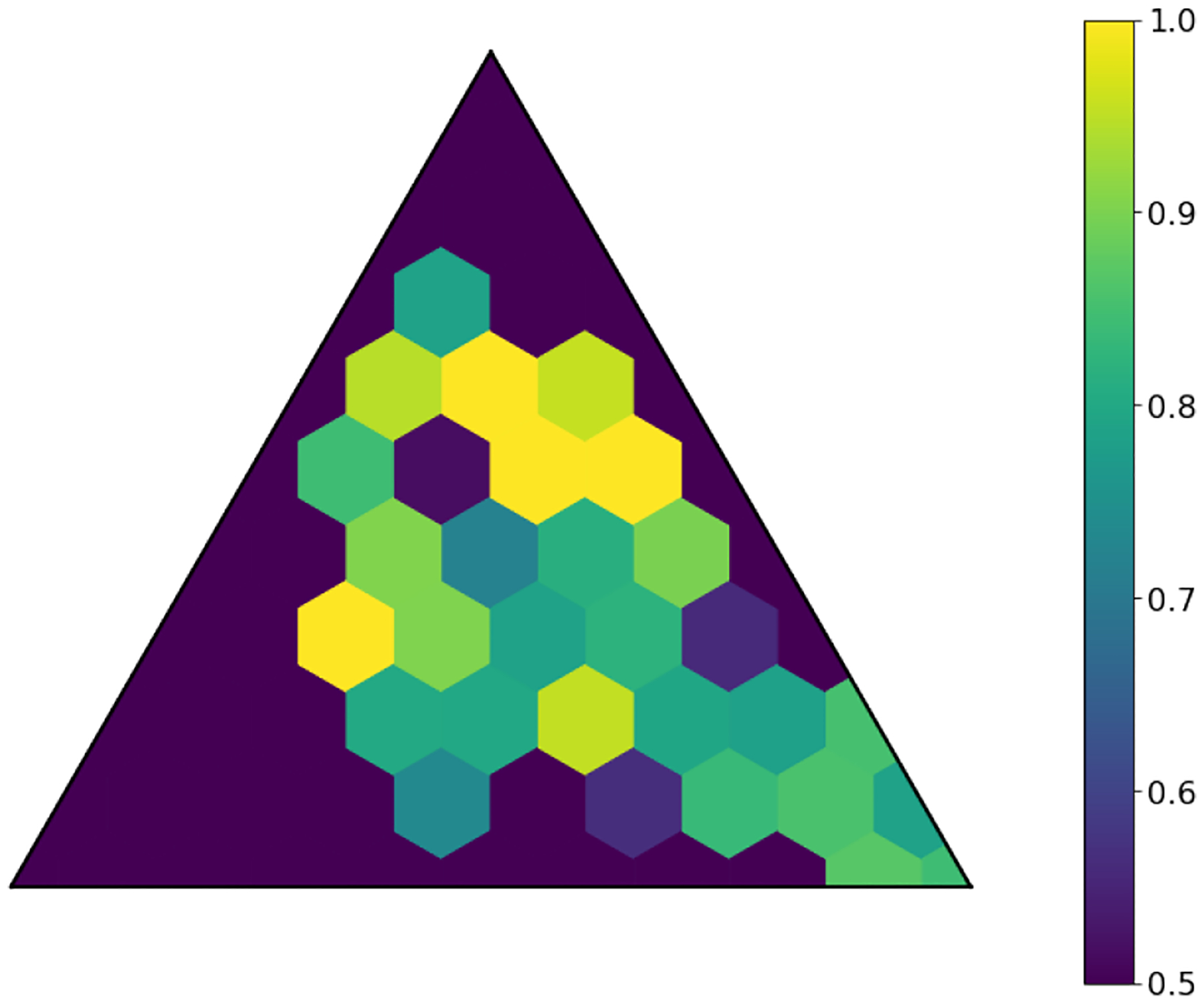
Local AUROCs of model with three experts and L2=0.01. Cells with insufficient subjects for scoring are set to 0.5.

**Fig. 10.**
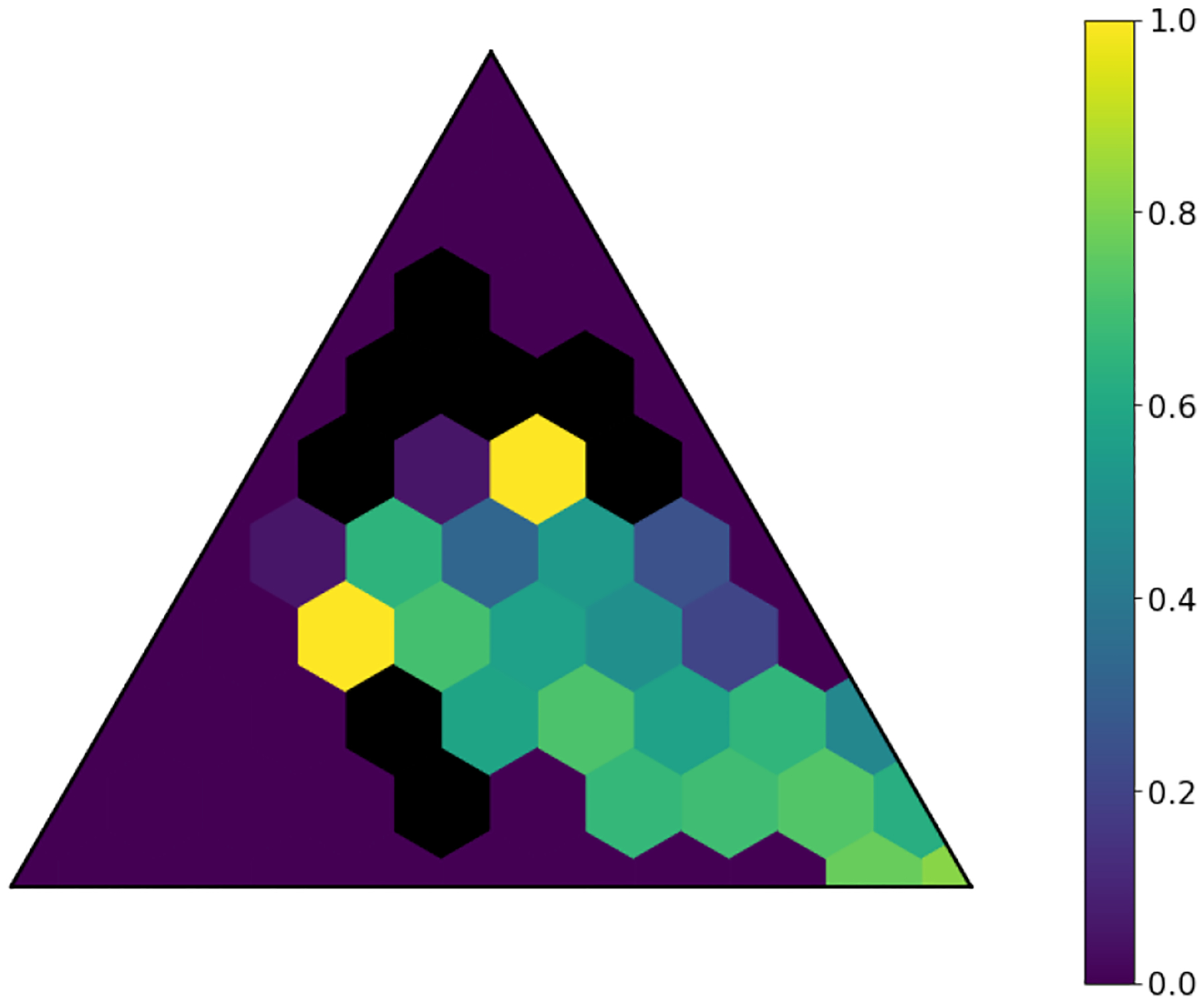
Local AUPRCs of model with three experts and L2=0.01. Cells with insufficient subjects for scoring are set to 0.

For the model with three experts, the distribution of patients among each fold is shown in Figure 7. All experts are shown. Cluster A is again the most heavily weighted cluster with 63% of assignments, with Clusters B and C near equal (Table 7). A representative fold from this model is shown in Figure 8. In this figure, true positives are once again found more often near Cluster A, while subjects more distributed throughout the other clusters exhibit lower overall risk. Local prediction performance is shown in Figures 9 and 10. Prediction quality can be seen to improve among patients who are more balanced between clusters.

## 5 LIMITATIONS AND FUTURE DIRECTIONS

The methods here describe phenotype discovery within a vast, observational clinical registry dataset. There are, however, limitations that exist. While showing individualized effect through phenotyping overcomes limitations present in propensity matching techniques, further analysis is needed for causal inference: This work is primarily used as hypothesis generation for future trial design. Further, the dataset itself is limited to structural data. While the data are separated in time to prior treatment and after treatment, the time-varying nature of risk is still not well represented: Electronic health record-based models will support more dynamic modeling of risk and of treatment decision making. As a result, there is the potential for unmeasured confounders to alter the phenotypic representations. Missing information—specifically about hemodynamic status and severity of shock—can be crucial, as it strongly influences the choice of therapy and is also strongly correlated with mortality. While the work here shows strong performance in modeling risk of mortality, thus potentially representing patient status well in the latent space of the model, it remains possible that additional data, not available, may improve risk performance, and in turn, jointly alter the phenotypic representation. This approach used the best available data, but future work within the electronic health record is necessary. Indeed, the findings here can potentially inform future, prospective studies that use data-driven clusters to better understand the benefits and risks of MCS devices in specific patient populations; these should be rigorous randomized control trials. Finally, the MoE approach described is regularized with respect to the number of clusters, but the number of clusters is pre-specified. An approach that discovers the number of clusters, accounting for limitations on small clusters and providing a variety of similarity metrics may help move beyond phenotyping toward more of a digital twin approach, based upon the vast quantities of data that could be made available both through the registry as well as electronic health record data. Further, MoEs have a chief limitation surrounding the type of data provided to each expert. The work here assumes that all data presented are clinically relevant risk factors that affect all subpopulations equally. However, if this is not the case, then methods that extract subtype-specific features are needed [14], because otherwise the model can become infinitely wide. This growth comes from combining all possible combinations of input data for downstream outcome prediction [35]. As a result, such MoEs are limited in their generalizability. They become particularly useful specifically in situations where a number of subpopulations are hypothesized, and the data provided to the experts is the same across these subtypes, or the distinct split across data modalities and how expert models are designed is clear. In these situations, each expert model can provide an estimate of outcome prediction with different feature weights without needing to architect specific expert models for each subset of clinical data available.

## 6 CONCLUSION

Identifying patient heterogeneity in observational clinical data may allow clinicians to understand both important phenotypes that have major adverse event outcomes for different driving risk factors as well as hypothesize different treatment strategies based upon individualized treatment effect estimation. However, risk estimation techniques alone are insufficient for this phenotype discovery, and matching techniques may suffer from limitations in relating the matching to addressable risk factors. This work sought to address these limitations by developing a deep mixture of experts approach that may both provide risk estimation and phenotype discovery. Applied to a clinical population of AMI-CS patients, this work both developed an accurate in-hospital mortality model as well as provided phenotyping of the AMI-CS patients and visualizations of cases where the prediction was confident versus regions in the latent space in which uncertainty may lead to false positive or false negative estimates. Through such interpretation, it then became possible to look at phenotypes of AMI-CS and more closely evaluate treatment effects through the choice of MCS device.

We have also shown that it is possible to explore the latent space of a deep MoE classifier to understand how it can aid in assigning archetypes. Rather than assigning to a single most representative cluster, this approach allows for a nuanced balancing and understanding of soft clustering. The outcomes-driven nature of the joint model training does strongly bias patients with the outcome of interest into one group. This allows for the model to separate patients by severity, with patients further from the strongest cluster exhibiting lower clinical risk. At the same time, the overall model performance does well further from this strong cluster membership. This approach shows that the deep MoE is successful and appropriate for separating heterogeneity in a clinical population and for separating those patients by disease severity.

## Figures and Tables

**Table 1.** Clinical Features Used in Study

Presentation Data	Treatment Data
Age	Need for Mechanical Support
Sex	Peak Troponin Value
Cardiac Arrest on First Contact	Requires Dialysis
Cardiac Arrest Prior to Hospitalization	Dialysis
Cardiac Arrest at Outside Clinical Facility	Atrial Fibrillation
Systolic Blood Pressure on First Contact	Ventricular Tachycardia Fibrillation
ST Elevation MI (STEMI)	Left Ventricular Ejection Fraction
Left Main Coronary Artery Stenosis	Cerebrovascular Accident
Proximal Left Anterior Descending Artery Stenosis	Length of Stay
Multi Vessel Disease	Other Vascular Complications Identified
Initial Creatinine Value	MCS Used or Not (medical therapy)
Initial Troponin Value	MCS Used: ECMO
—	MCS Used: IABP
—	MCS Used: LVAD
—	MCS Used: Other

**Table 2.** Clinical Dataset Mortality AUROC Values for All Models with L2 Regularization = 0.01

	Baseline	2 Experts	4 Experts	5 Experts	6 Experts	8 Experts	10 Experts
XGBoost	**0.880** ± **0.006**						
Logistic Regression	0.839 ± 0.004						
Small Model		**0.843** ± **0.011**	**0.851** ± **0.006**	**0.848** ± **0.009**	**0.851** ± **0.004**	**0.849** ± **0.008**	**0.848** ± **0.005**
Mid-Sized Model		0.841 ± 0.013	0.846 ± 0.011	0.842 ± 0.003	0.843 ± 0.007	0.841 ± 0.008	0.840 ± 0.011
Large Model		0.763 ± 0.098	0.822 ± 0.012	0.814 ± 0.009	0.812 ± 0.006	0.810 ± 0.013	0.818 ± 0.017
Later Hidden Connection		0.626 ± 0.132	0.559 ± 0.108	0.688 ± 0.134	0.701 ± 0.107	0.756 ± 0.097	0.645 ± 0.115
No Hidden Connection		0.618 ± 0.115	0.755 ± 0.040	0.722 ± 0.054	0.777 ± 0.007	0.712 ± 0.071	0.729 ± 0.079

Reprinted, with permission, from Reference [16]. ©2021 IEEE.

**Table 3.** Clinical Dataset Mean Pairwise ARI Then AUROC Values (and 95% Confidence Interval) for All Models with Varied L2 Regularization for the Best-performing Model and Varied Number of Experts

Metric	L2 Penalty	2 Experts	3 Experts	4 Experts	5 Experts	6 Experts	10 Experts
Pairwise ARI	0.1	0.349 ± 0.004	0.351 ± 0.001	0.349 ± 0.002	0.349 ± 0.003	0.351 ± 0.005	0.352 ± 0.004
Pairwise ARI	0.001	0.000 ± 0.000	0.000 ± 0.000	0.000 ± 0.000	0.000 ± 0.000	0.000 ± 0.000	0.201 ± 0.128
Pairwise ARI	0.002	0.137 ± 0.110	−0.002 ± 0.003	0.120 ± 0.129	0.167 ± 0.104	0.225 ± 0.127	0.339 ± 0.081
Pairwise ARI	0.004	0.105 ± 0.103	0.414 ± 0.083	0.468 ± 0.094	0.430 ± 0.037	0.574 ± 0.043	0.446 ± 0.079
Pairwise ARI	0.006	0.438 ± 0.046	0.387 ± 0.121	0.307 ± 0.159	0.602 ± 0.057	0.398 ± 0.082	0.628 ± 0.022
Pairwise ARI	0.008	0.442 ± 0.062	0.220 ± 0.096	0.296 ± 0.068	0.510 ± 0.071	0.409 ± 0.067	0.208 ± 0.077
Pairwise ARI	0.01	0.425 ± 0.040	0.195 ± 0.068	0.239 ± 0.089	0.433 ± 0.059	0.145 ± 0.040	0.186 ± 0.021
Pairwise ARI	0.05	0.007 ± 0.007	0.021 ± 0.018	0.050 ± 0.017	0.049 ± 0.011	0.056 ± 0.013	0.094 ± 0.012
Pairwise ARI	0.1	0.007 ± 0.006	0.012 ± 0.007	0.005 ± 0.005	0.042 ± 0.009	0.029 ± 0.010	0.046 ± 0.012
AUROC	0.001	0.839 ± 0.002	0.841 ± 0.001	0.840 ± 0.001	0.840 ± 0.005	0.843 ± 0.002	0.842 ± 0.003
AUROC	0.002	0.840 ± 0.004	0.839 ± 0.003	0.841 ± 0.004	0.842 ± 0.001	0.844 ± 0.003	0.843 ± 0.003
AUROC	0.004	0.844 ± 0.002	0.848 ± 0.003	0.849 ± 0.003	0.846 ± 0.002	0.846 ± 0.003	0.847 ± 0.001
AUROC	0.006	0.850 ± 0.004	0.846 ± 0.004	0.846 ± 0.002	0.849 ± 0.002	0.850 ± 0.002	0.847 ± 0.002
AUROC	0.008	0.850 ± 0.003	0.850 ± 0.003	0.850 ± 0.003	0.850 ± 0.002	0.847 ± 0.005	0.849 ± 0.004
AUROC	0.01	0.848 ± 0.003	0.849 ± 0.003	0.852 ± 0.002	0.851 ± 0.003	0.846 ± 0.002	0.848 ± 0.002
AUROC	0.05	0.839 ± 0.002	0.842 ± 0.002	0.845 ± 0.005	0.849 ± 0.003	0.852 ± 0.004	0.855 ± 0.002
AUROC	0.1	0.839 ± 0.003	0.839 ± 0.001	0.842 ± 0.007	0.846 ± 0.004	0.846 ± 0.004	0.850 ± 0.006

We show a sample of experts beyond 5 but all saw a monotone decrease from 6 and up.

**Table 4.** Clinical Dataset Mean AUPRC, Uncertainty, Resolution, and Reliability (and 95% Confidence Interval) for All Models with Varied L2 Regularization for the Best-performing Model and Varied Number of Experts

Metric	L2 Penalty	2 Experts	3 Experts	4 Experts	5 Experts	6 Experts	10 Experts
AUPRC	0.001	0.723 ± 0.003	0.723 ± 0.002	0.724 ± 0.002	0.723 ± 0.004	0.726 ± 0.003	0.725 ± 0.004
AUPRC	0.002	0.723 ± 0.003	0.723 ± 0.002	0.723 ± 0.005	0.721 ± 0.005	0.726 ± 0.002	0.728 ± 0.002
AUPRC	0.004	0.725 ± 0.002	0.729 ± 0.003	0.728 ± 0.002	0.727 ± 0.003	0.727 ± 0.003	0.728 ± 0.002
AUPRC	0.006	0.730 ± 0.002	0.727 ± 0.001	0.727 ± 0.003	0.730 ± 0.002	0.730 ± 0.002	0.727 ± 0.004
AUPRC	0.008	0.729 ± 0.004	0.730 ± 0.004	0.732 ± 0.002	0.728 ± 0.001	0.727 ± 0.006	0.730 ± 0.003
AUPRC	0.01	0.727 ± 0.004	0.730 ± 0.004	0.732 ± 0.004	0.729 ± 0.005	0.727 ± 0.003	0.730 ± 0.004
AUPRC	0.05	0.725 ± 0.003	0.727 ± 0.002	0.730 ± 0.005	0.732 ± 0.002	0.733 ± 0.002	0.735 ± 0.003
AUPRC	0.1	0.722 ± 0.002	0.727 ± 0.001	0.728 ± 0.005	0.729 ± 0.004	0.730 ± 0.003	0.733 ± 0.002
Resolution	0.001	1.355 ± 0.007	1.353 ± 0.008	1.365 ± 0.006	1.357 ± 0.011	1.367 ± 0.012	1.353 ± 0.007
Resolution	0.002	1.364 ± 0.005	1.359 ± 0.004	1.359 ± 0.008	1.356 ± 0.005	1.353 ± 0.003	1.367 ± 0.006
Resolution	0.004	1.356 ± 0.006	1.351 ± 0.008	1.359 ± 0.014	1.362 ± 0.006	1.362 ± 0.010	1.361 ± 0.008
Resolution	0.006	1.357 ± 0.005	1.357 ± 0.013	1.356 ± 0.005	1.356 ± 0.006	1.353 ± 0.011	1.351 ± 0.004
Resolution	0.008	1.348 ± 0.008	1.352 ± 0.006	1.357 ± 0.004	1.346 ± 0.006	1.347 ± 0.003	1.362 ± 0.009
Resolution	0.01	1.342 ± 0.002	1.348 ± 0.010	1.354 ± 0.008	1.346 ± 0.007	1.355 ± 0.008	1.345 ± 0.005
Resolution	0.05	1.349 ± 0.009	1.339 ± 0.007	1.346 ± 0.012	1.345 ± 0.008	1.345 ± 0.015	1.346 ± 0.011
Resolution	0.1	1.345 ± 0.009	1.352 ± 0.003	1.346 ± 0.013	1.343 ± 0.009	1.349 ± 0.011	1.350 ± 0.013
Reliability	0.001	0.350 ± 0.003	0.350 ± 0.003	0.353 ± 0.002	0.350 ± 0.004	0.355 ± 0.005	0.348 ± 0.002
Reliability	0.002	0.353 ± 0.002	0.352 ± 0.001	0.351 ± 0.003	0.351 ± 0.002	0.349 ± 0.001	0.354 ± 0.002
Reliability	0.004	0.350 ± 0.003	0.348 ± 0.003	0.352 ± 0.005	0.353 ± 0.003	0.352 ± 0.003	0.354 ± 0.003
Reliability	0.006	0.353 ± 0.002	0.351 ± 0.004	0.350 ± 0.003	0.352 ± 0.003	0.350 ± 0.004	0.350 ± 0.001
Reliability	0.008	0.350 ± 0.004	0.352 ± 0.002	0.353 ± 0.001	0.348 ± 0.002	0.348 ± 0.003	0.354 ± 0.003
Reliability	0.01	0.349 ± 0.001	0.350 ± 0.004	0.352 ± 0.003	0.349 ± 0.003	0.353 ± 0.002	0.348 ± 0.002
Reliability	0.05	0.350 ± 0.003	0.347 ± 0.004	0.349 ± 0.003	0.350 ± 0.003	0.350 ± 0.004	0.350 ± 0.004
Reliability	0.1	0.349 ± 0.004	0.351 ± 0.001	0.349 ± 0.002	0.349 ± 0.003	0.351 ± 0.005	0.352 ± 0.004

We show a sample of experts beyond 5 but all saw a monotone decrease from 6 and up.

**Table 5.** Clinical Cluster Characteristics

Cluster	A	B	C	D	E
Number	21,728	2,809	2,053	1,639	75
Age (SD)	68.2 (11.8)	54.9 (9.4)	56.1 (7.5)	52.3 (9.5)	46.0 (7.5)
Male	61.1%	92.5%	81.4%	81.8%	97.3%
Smoker	31.0%	48.7%	55.1%	52.5%	44.0%
STEMI	81.5%	80.99%	71.0%	55.1%	97.3%
MD	56.8%	71.09%	19.5%	48.8%	18.7%
Urgent PCI	11.0%	11.8%	19.8%	30.7%	0.0%
Emergent PCI	78.8%	82.5%	75.4%	64.4%	93.3%
Mortality	30.3%	14.6%	7.5%	7.3%	10.7%
Only IABP	30.3%	35.2%	22.7%	25.1%	22.7%
Only LVAD	6.5%	7.7%	3.4%	4.3%	1.3%
No MCS Device	56.7%	49.1%	68.4%	65.3%	75.3%

Age is represented as mean and standard deviation (SD). STEMI = ST Elevation Myocardial Infarction. MD = Multivessel Disease. Reprinted, with permission, from Reference [16]. ©2021 IEEE.

**Table 6.** Per-cluster Mortality Rate (and 95% Confidence Interval)

Cluster	Entire Group	Only IABP	Only LVAD	None
A	30.3 (29.7–30.9)	33.9 (32.7–35.0)	49.8 (47.1−52.4)	24.3 (23.5–25.0)
B	14.6 (13.3–16.0)	14.5 (12.3–16.8)	29.6 (23.6–36.2)	8.6 (7.2–10.2)
C	7.5 (6.4–8.7)	10.5 (7.9–13.7)	25.7 (16.0–37.6)	4.2 (3.2–5.4)
D	7.3 (6.1–8.6)	7.8 (5.4–10.8)	22.9 (13.7–34.4)	5.0 (3.7–6.4)
E	10.7 (4.7–19.9)	11.8 (1.5–36.4)	0.0 (0.0–97.5)	9.1 (3.0–20.0)
All	25.7 (25.2–26.2)	29.0 (28.1–30.0)	45.2 (42.9–47.6)	19.9 (19.3–20.5)

Gray values reflect those groups for which there is not a significant difference between that group and the corresponding group in the total population. Reprinted, with permission, from Reference [16]. ©2021 IEEE.

**Table 7.** Weight Contribution of Each Cluster

Cluster	5 Experts	3 Experts
A	77.0%	62.9%
B	12.2%	20.0%
C	8.5%	17.2%
D	2.1%	—
E	0.09%	—

As only three clusters can be visualized at once, this table aids in assessing impact of truncation.

## CCS Concepts:

- Applied computing → Health care information systems; Health informatics;
- Computing methodologies → Ensemble methods; *Regularization*; *Feature selection;*

## Footnotes

1 Confidence intervals in this table are calculated by the Clopper-Pearson method. This method is notable for producing conservative estimations (potentially too large), but its coverage is guaranteed to be at least 1-*α* (here, 95%).
